# Do Ergogenic Aids Alter Lower Extremity Joint Alignment During a Functional Movement Lunge Prior to and Following an Exercise Bout?

**DOI:** 10.1515/hukin-2015-0002

**Published:** 2015-04-07

**Authors:** Chris Mills, James Knight, Gemma Milligan

**Affiliations:** 1 Department of Sport and Exercise Science, Spinnaker Building, University of Portsmouth, UK.

**Keywords:** tape, injury, stability

## Abstract

Ergogenic aids have been used to alter joint kinematics in an attempt to minimise injury risk, yet the effectiveness of these aids may be compromised following a bout of exercise. This preliminary study aimed to measure the effect of compression garments and Kinesio Tape® on lower extremity joint alignment prior to and following an exercise bout. Eight male athletes (age = 24.1 ± 3.0 years, body height = 177.4 ± 5.2 cm, body mass = 72.3 ± 7.2 kg) volunteered to participant in this study. Joint kinematics were recorded whilst all participants performed three rotational lunges, in three conditions (control, compression garment, Kinesio Tape®), prior to and following a 10 minute exercise bout. Frontal plane kinematics (lateral pelvic tilt, knee valgus, ankle inversion/eversion) were used to assess ergogenic aid effectiveness during the lunge. Participants exhibited no significant differences in joint kinematics between ergogenic aid conditions prior to the exercise bout. Following exercise the only significant difference occurred within the Kinesio Tape® condition where maximum knee valgus angle significantly increased from 6.5° prior to exercise, to 7.7° following the exercise bout. The results of this study suggest joint kinematics are not affected by the ergogenic aids in this study prior to an exercise bout. However, there is evidence to suggest that the application of Kinesio Tape® may allow an increase in knee valgus angle following a bout of exercise, yet, compression garments are effective at maintaining joint alignment following a bout of exercise.

## Introduction

A movement matrix is the integration of structural and functional properties of the neuromuscular system that combine to produce efficient movement (Cook, 2009). Weak links within this matrix consequently produce forces that cause possibly injurious kinematics and joint misalignment (Cook, 2009). A lunge has been proposed as one exercise that can be used as an effective screening tool to assess the risk of lower extremity injury through misaligned joint kinematics (Kritz et al., 2009). The lunging movement is utilised when decelerating and cutting, and when performed incorrectly the occurrence of injury is greatly increased (Cook, 2009). Misaligned joint kinematics at the hip, knee and ankle are associated with a greater potential risk of injury during a poorly performed lunge motion (Kritz et al., 2009). Correct lunging kinematics should include a level pelvis (in the frontal plane) to avoid lateral pelvic tilt (Powers, 2010; Chang et al., 2005). Additionally knee valgus/varus should be minimised to avoid injury and ankle eversion/inversion should be reduced to sustain the body in equilibrium by maintaining the centre of mass within the support base limits (Thijs et al., 2007; Boyle, 2010; Kritz et al., 2009; Shumway-Cook and Woollacott, 2001).

Ergogenic aids such as traditional taping are used as an immediate temporary solution to maintain or improve joint alignment and kinematics (Bennell and Goldie, 1994; Lin et al., 2010; Fleet et al., 2009; Palmer and Lane-Larsen, 1994; Cook et al., 2006). However, some investigations have found that the effectiveness and durability of traditional taping was reduced after 10 minutes (Rarick et al., 1962; Fleet et al., 2009). Kinesio Tape® came to prominence in the 2008 Beijing Olympic Games and unlike traditional tape it is designed to stretch 55–60% of its original length and to contour the skin for up to 5 days (Williams et al., 2012; Kase et al., 2003). Kinesio Tape® creates extra resistance by applying tension across the joint surface, and furthermore triggering increased muscular activation (Kase et al., 2003; Yoshida and Kahanov, 2007), this may enable the wearer to maintain or improve joint alignment during a lunge for an extended period of time (> 10 min) or even after a bout of exercise. Furthermore, previous research proposed increased limb stability, corrected body alignment during biomechanical movements, increased neuromuscular control (Williams et al., 2012; Huang et al., 2011; Yoshida and Kahanov, 2007; Hsu et al., 2009; Callaghan, 2002; Lin et al., 2010) and reduced injury (Fu et al., 2008), following taping. In contrast, research has also suggested that Kinesio Tape® only reduces the misalignment of joint kinematics during non-weight bearing Functional Movement Screen (FMS) movements (An et al., 2012) and Kinesio Tape® does not affect lower limb mechanics pre and post 12 hours of exercise (Lins et al., 2012; Fu et al., 2008). Conflicting research exists on the effects of Kinesio Tape® on its potential for maintaining or improving joint alignment, thus this study could provide a further insight into its effectiveness pre and post a short bout of exercise.

Compression garments (CG) are another popular ergogenic aid that may increase stability, motor function, movement and control (Rennie et al., 2000; Pearce et al., 2009). Particular CG have integrated support web technology, claimed to mechanically support muscles and joints causing increased compression and functional control (cw-x.com, n.d.). Benefits of wearing CG have been reported as increasing pelvic stability and enhanced functional motor control through neurological improvements (Rennie et al., 2000; Doan et al., 2003; Pearce et al., 2009). The compressive nature of the garment may have enhanced skin tension resulting in increased muscular activity (Yoshida and Kahanov, 2007). Therefore, it is possible that CG leggings covering the hips, knees and ankles may be used to maintain joint alignment during a lunge and their effectiveness may not be compromised following an exercise bout. Wearing either Kinesio Tape® or CGs may also improve joint proprioception as applying pressure and a stretch to the skin can stimulate cutaneous mechanoreceptors leading to sensory feedback of joint movement and position (Halseth et al., 2004).

Ergogenic aids have been used to improve or maintain joint kinematics in an attempt to minimise the injury risk associated with segment misalignment and this study could provide a further insight into the effectiveness of these aids pre and post a short bout of exercise. The aim of this preliminary study was to measure the effects of compression garments and Kinesio Tape® on lower extremity joint alignment prior to and following an exercise bout. Firstly, it was hypothesised that there would be a significant difference in lower extremity joint alignment between ergogenic aids and the control condition prior to an exercise bout. Secondly, it was hypothesised that there would be a significant difference in lower extremity joint alignment within each ergogenic aid prior to and following an exercise bout.

## Material and Methods

### Participants

Following institutional favourable ethical opinion eight male participants with no existing lower extremity injuries were recruited for this study. All participants gave written informed consent to participate. The participants’ mean ± SD age was 24.1 ± 3.0 years, body mass 72.3 ± 7.2 kg, and height 177.4 ± 5.2 cm. In addition the lunge length (88.8 ± 5.1 cm) was standardised to equal the distance from the great trochanter to the floor (Farrokhi et al., 2008).

### Procedures

Following a warm up which consisted of a 5 min low intensity treadmill run and lower extremity stretches, fourteen retro-reflective markers (5 mm radius) were positioned on predetermined anatomical landmarks (Picture 1). Additionally, the height from the ground to the participant’s greater trochanter was measured and used to standardize the participant’s lunge length (Farrokhi et al., 2008). The participants (n=8) were randomly assigned to one of the ergogenic aids (Kinesio Tape® or CG) or the control condition. Kinesio Tape® was applied following the instructions stated by Kase et al. (2003). This was applied to the gluteus medius, patella tracking, tibialis anterior and peroneus longus. The CG used a pair of CW-X® Support Web™ compression leggings (medium) in accordance with the manufacturer’s fitting recommendations (height 170–186 cm), which covered the participants’ pelvis, knees and upper ankles. The participants performed all three ergogenic aid conditions without shoes. Twelve Oqus Cameras (Qualisys Oqus 300/310, Sweden), with a sampling frequency of 100 Hz, were placed in an arc around the activity volume and used to track the positional coordinates of the markers. The activity volume was calibrated using a calibration wand and L-shaped frame (Qualisys, Sweden) and the mean accuracy of the camera system was 0.4 mm. Electrical tape marking the lunge direction was positioned along the x axis identifying the sagittal plane. The laboratory coordinate system identified x as the anteroposterior line, y as mediolateral and z as vertical.

The participants were asked to perform three rotational lunges and were instructed to step forward with their dominant leg along the x axis and lower their trunk until their non-dominant knee was at a distance of approximately 3 cm above the ground, before returning to their starting position. They were also asked to keep an upright torso and to rotate their torso to the side of the lead lower extremity (Kritz et al., 2009; Cook, 2003). The participants then completed a 10-minute treadmill run exercise bout at 70% of their perceived maximum intensity (Nolan and Kennedy, 2009; Fleet et al., 2009). Post exercise, they were asked to repeat the three rotational lunges. Following completion of the test, the participants were asked to rest and when ready, the test procedure was repeated for each of the remaining ergogenic aid conditions, ensuring each participant ran at the same speed (percentage of maximum intensity) as in their previous ergogenic aid condition.

Markers were identified in Qualisys Track Manager (2.6, Qualisys Track Manager, Sweden), any gaps in the trajectories were linearly interpolated, and the data were filtered using a second order low pass Butterworth filter with a cut off of 10 Hz. The processed data were then used to calculate the study variables; maximal lateral pelvic tilt, maximal knee valgus and maximal ankle eversion/inversion during each lunge. The maximum lateral pelvic tilt angle was calculated between the ipsilateral and contralateral anterior superior iliac spine (ASIS) relative to the XY plane. The maximal knee valgus angle was calculated between the ipsilateral ASIS and patella relative to the XZ plane. The maximal ankle eversion (+) and inversion (−) angle was calculated between the angle between the upper and lower calcaneus (ipsilateral) in the XZ plane. The maximum angles were reported between the dominant leg’s foot touching (initial contact) and breaking contact with the ground again following the lunge. All parameters were averaged from the three lunges.

### Statistical Analyses

Lower extremity kinematics were statistically analysed using PASW software (Version 18) and all data were checked for normality (Shapiro-Wilk test). A repeated measures ANOVA was implemented to assess differences across the three ergogenic aids prior to the exercise bout. Post hoc testing, consisting of independent t-tests with a Bonferroni correction (p < .017), were performed when the ANOVA identified any significant interactions. A parametric paired samples *t*-test was used to examine the differences prior to and following the exercise bout within each ergogenic aid condition. Effect sizes (partial eta squared η^2^ and Cohen’s *d*) were reported for results, where appropriate. Parametric effect sizes were defined as large *d* > 0.8, moderate as between 0.8 and 0.5, and a small effect size defined as < 0.5 (Cohen, 1988).

## Results

### Maximal lateral pelvic tilt prior to an exercise bout

**Figure 1:**
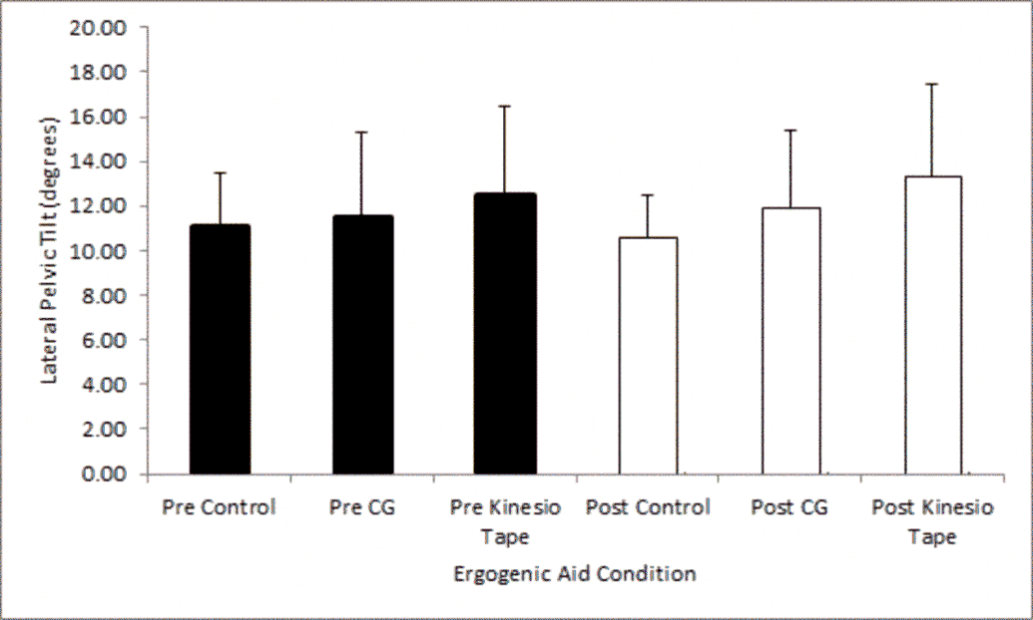
Maximum lateral pelvic tilt pre and post an exercise bout

The greatest lateral pelvic tilt was found in the Kinesio Tape® condition (12.6°±3.9), followed by the CG (11.6°±3.7) and the control (11.1°±2.4) (Figure 1), however, there were no significant differences between ergogenic aid conditions (F_(2,14)_ =0.952; *p*=0.410, η^2^ =0.120).

### Maximal knee valgus prior to an exercise bout

**Figure 2:**
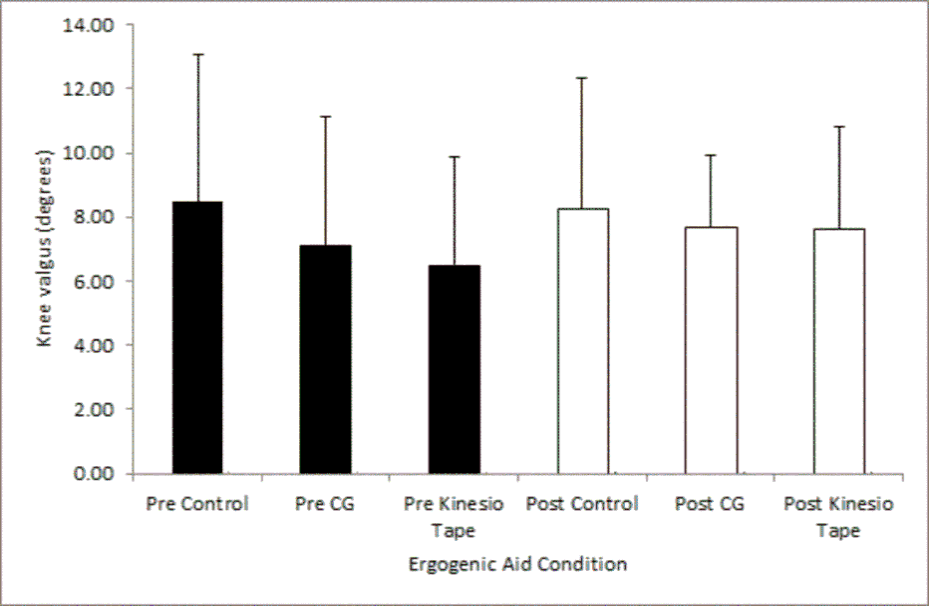
Maximum knee valgus angles during a lunge pre and post an exercise bout

The greatest knee valgus angle was found in the control condition (8.5°±4.6), followed by the CG (7.1°±4.0) and the Kinesio Tape® condition (6.5°±3.3) (Figure 2), however, there was no significant difference between ergogenic aid conditions (F_(2,14)_ =3.238; *p*=0.070, η^2^ =0.316).

### Maximal ankle eversion / inversion prior to an exercise bout

**Figure 3:**
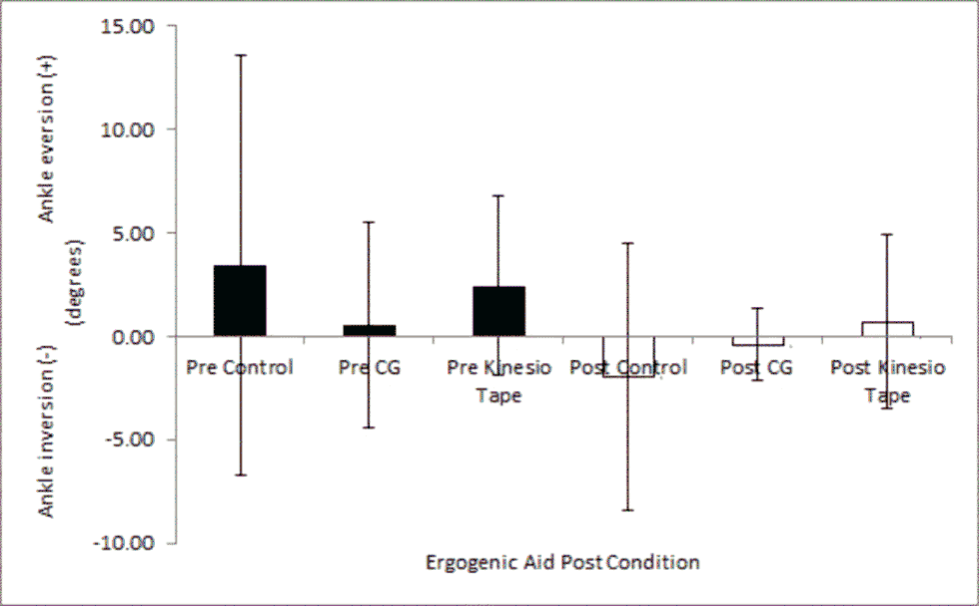
Maximum ankle inversion / eversion angles during a lunge pre and post an exercise bout

The least maximal ankle eversion (0.5°) occurred in the compression garment condition (Figure 3), however, there was no significant difference between ergogenic aid conditions prior to an exercise bout (F_(2,14)_=0.306; *p*=0.741, η^2^ =0.042). This finding was similar for maximum ankle inversion, with the Kinesio Tape® condition exhibiting the least amount of ankle inversion (Figure 3), however, no significant differences were found between ergogenic aid conditions (F_(2,14)_=0.816; *p*=0.462, η^2^ =0.104).

### Maximal lateral pelvic tilt prior to and following an exercise bout

Results indicated minimal changes in maximum lateral pelvic tilt following a bout of exercise (Figure 1) with no significant differences within each ergogenic aid condition (Kinesio Tape®: *t* =−.858, *p*=0.419, *d*=0.11), (CG: t =−1.036, *p*=0.335, *d*=0.19). The control condition also showed no significant differences (t = 1.189, *p*=0.273, *d*=0.25) following a bout of exercise.

### Maximal knee valgus prior to and following an exercise bout

Following an exercise bout maximal knee valgus significantly increased by 1.2° (Figure 2) within the Kinesio Tape® condition (*t* =−2.611, *p*=0.035, *d*=0.35) possibly indicating a reduction in the support provided by the tape following exercise. However, there were no significant differences in maximum knee valgus within the CG condition (*t* =−.597, *p*=0.569, *d*=0.17) or the control condition (*t* =.308, *p*=0.767, *d*=0.05) following an exercise bout.

### Maximal ankle eversion / inversion prior to and following an exercise bout

Maximal ankle eversion did not significantly change following an exercise bout within the Kinesio Tape® (*t* = 1.388, *p*=0 .208, *d*=0.40), CG (*t* =.536, *p*=0 .608, *d*=0.25) and control (*t* = 1.216, *p*=0.264, *d*=0.64) conditions. Similarly, maximal ankle inversion was not significantly different following an exercise bout, CG (*t* = 0.152, *p*=0.884, *d*=0.07), Kinesio Tape® (*t* =1.062, *p*=0.324, *d*=0.24), control (*t* = −0.032, *p*=0.975, *d*=0.01).

## Discussion

This preliminary study aimed to measure the effects of compression garments and Kinesio Tape® on lower extremity joint kinematics during a lunge prior to and following an exercise bout. Key findings indicated that lower extremity joint alignment remained unaffected with the application of any of the ergogenic aids prior to exercise, suggesting that these aids were not required as a preventative measure for athletes without a pre-existing lower extremity injury. However, following a bout of exercise there is evidence to suggest that the application of Kinesio Tape® may increase knee joint misalignment and hence, caution is advised as taping may increase the risk of musculoskeletal knee injuries, if worn for more than 10 minutes of exercise.

Lower extremity joint kinematics were similar between ergogenic aid conditions prior to an exercise bout suggesting that the application of Kinesio Tape® or compression garments were not required as a preventative measure to correct or maintain joint alignment, rejecting hypotheses one. The effectiveness of tape and compression garments as an immediate temporary solution to improve kinematics (Bennell and Goldie, 1994; Lin et al., 2010; Fleet et al., 2009; Palmer and Lane-Larsen, 1994; Cook et al., 2006) during a lunge was not supported during this preliminary study. Despite these findings, it is recommended that any potential positive psychological benefit must also be considered as research has indicated that the ‘sham’ effect can also produce a positive effect on joint kinematics and reduce pain (Franettovich et al., 2008; Pruscino and Halson, 2013). A sample group with pre-existing lower extremity functional joint misalignment may still benefit from the combination of improved proprioception (Halseth et al., 2009), and the perceived psychological benefits, from the ergogenic aids, and improve their lower extremity kinematics during a lunge movement prior to an exercise bout.

The findings of this study also suggest that compression garments do not have an effect on lower extremity joint alignment following a bout of exercise; however, there is evidence to suggest that the application of Kinesio Tape® alters lower extremity knee kinematics. Results demonstrated that the maximum knee valgus angle increased significantly by 1.2° following the exercise bout, partially accepting hypotheses two. When interpreting these results, it is important to consider the magnitude of the knee valgus increase reported (∼18%), although statistically significant, is a change in knee valgus of 1.2° important in terms of injury reduction. Published literature suggests that anterior cruciate ligament rupture was generally observed when knee valgus angles exceeded 12° (Koga et al., 2010). Furthermore, an increase in knee valgus of 7.6° has also been shown to differentiate between injured and non-injured female athletes (Hewett et al., 2005). Therefore, an increase of 1.2° in knee valgus following an exercise bout may indicate increased joint misalignment and injury risk.

Previous research has stated that the effectiveness of traditional taping is compromised after 10 minutes (Rarick et al., 1962; Fleet et al., 2009). Although published research suggests Kinesio Tape® may be beneficial in the short term to correct body alignment and improve the joint range of motion during sporting movements (Williams et al., 2012), following an exercise bout its effectiveness is also compromised, similar to traditional tape. The reason for this increase in knee valgus may be firstly linked to the degeneration of the elastic properties of the Kinesio Tape® following exercise; this may compromise the tension across the joint and the proprioceptive response. However, Kinesio Tape® is designed to stretch 55–60% of the original length to contour the skin for up to 5 days (Williams et al., 2012; Kase et al., 2003). Therefore, tape degeneration is unlikely, suggesting another mechanism at work. It may be possible that the participant places a higher reliance on the tape after the exercise bout, in essence allowing the tape to control/limit the knee kinematics rather than a conscious neuromuscular refinement of the motor task based proprioception. Similar findings have been reported for patellar tape where the application of tape sometimes worsened the joint position sense of the participants (Callaghan et al., 2002). Future work could investigate the role of proprioception in joint control following repeated exercise bouts to understand this kinematic response whilst wearing Kinesio Tape®.

The application of these results beyond the sample of athletes without self-reported preexisting lower extremity injuries should be treated with caution. Whilst these results suggest that compression garments or Kinesio Tape® do not alter lower extremity joint kinematics during a lunge prior to an exercise bout, and therefore, are not an effective ergogenic aid for injury prevention, it is important to investigate their effectiveness within a population sample with a pre-existing lower extremity injury. Additionally, it is also important to note that following an exercise bout, Kinesio Tape® can increase knee valgus kinematics in a sample group without preexisting lower extremity injury; therefore, this may increase the risk of further injury in a group with a pre-existing lower extremity injury. Although these findings warrant further investigation with participants with a pre-existing lower extremity injury, caution must be used in order to minimise further injury during testing. Importantly, this study has highlighted that compression garments are effective at maintaining lower extremity joint kinematics following a bout of exercise and could be recommended as an alternative to taping to minimise injury risk.

## Conclusions

The results of this study suggest that lower extremity joint alignment, during a lunge, is not affected by the ergogenic aids in this study prior to an exercise bout. Furthermore, there is evidence to suggest that the application of Kinesio Tape® may increase knee valgus following a bout of exercise. Caution is advised as taping may increase the risk of musculoskeletal knee injuries if worn for more than 10 minutes of exercise. Compression garments are effective at maintaining lower extremity joint kinematics following a bout of exercise and could be recommended as an alternative to taping to minimise injury risk.

## Figures and Tables

**Picture 1:**
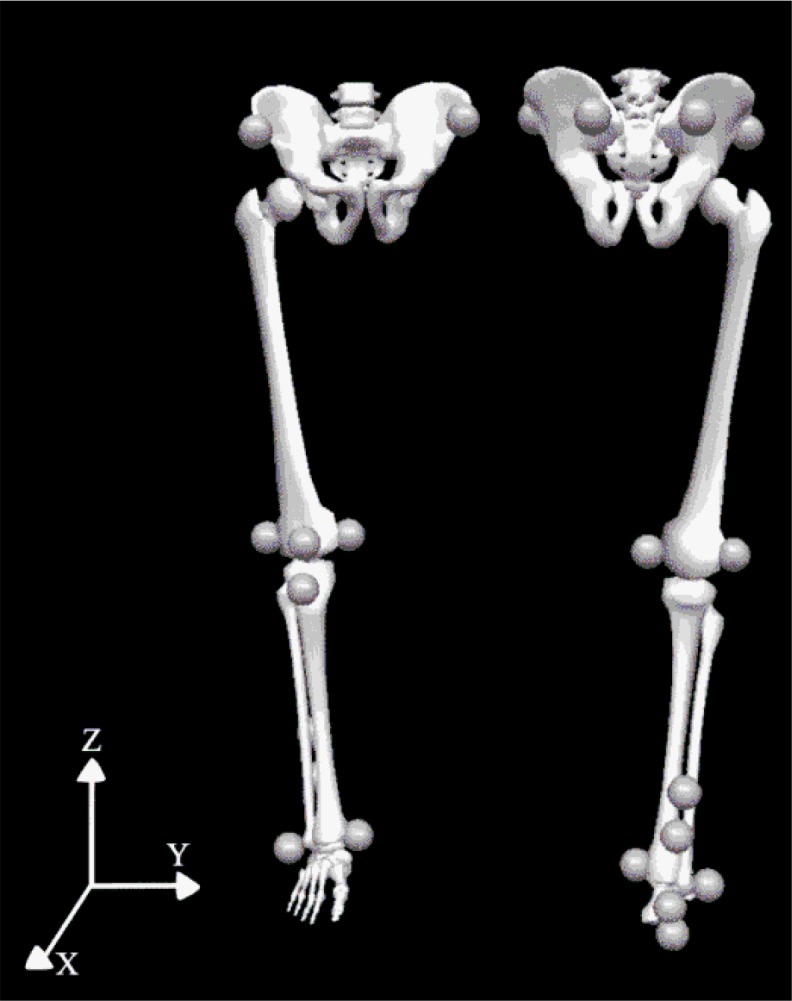
Marker locations (based on Ferber and Benson, 2011; Floyd, 2012; Thijs et al., 2012) on the lower extremity (right leg) with a global coordinate system.
